# Influence of phosphorylation on intermediate filaments

**DOI:** 10.1515/hsz-2023-0140

**Published:** 2023-04-20

**Authors:** Julia Kraxner, Sarah Köster

**Affiliations:** Max Delbrück Center for Molecular Medicine, Robert-Rössle-Straße 10, D-13125 Berlin, Germany; German Centre for Cardiovascular Research (DZHK), Partner Site Berlin, D-10785 Berlin, Germany; University of Göttingen, Institute for X-Ray Physics, Friedrich-Hund-Platz 1, D-37077 Göttingen, Germany; German Center for Cardiovascular Research (DZHK), Partner Site Göttingen, D-37075 Göttingen, Germany

**Keywords:** assembly and disassembly, cytoskeleton, mechanics, mitosis, posttranslational modification, vimentin

## Abstract

The cytoskeleton of eukaryotes consists of actin filaments, microtubules and intermediate filaments (IF). IFs, in particular, are prone to pronounced phosphorylation, leading to additional charges on the affected amino acids. In recent years, a variety of experiments employing either reconstituted protein systems or living cells have revealed that these altered charge patterns form the basis for a number of very diverse cellular functions and processes, including reversible filament assembly, filament softening, network remodeling, cell migration, interactions with other protein structures, and biochemical signaling.

## 1 Introduction

In addition to biological and biochemical functions, biological cells possess a variety of mechanical and dynamic properties, which enable them to divide, contract, or move. The so-called cytoskeleton, a complex biopolymer network composed of actin filaments, microtubules and intermediate filaments (IFs) lends the cell its intriguing viscoelasticity. The three filament types, each of which contributes distinct characteristics to the cytoskeleton, are complemented by passive cross-linker proteins and active molecular motors (Huber et al. 2015). For each cell type, the specific composition and architecture of the cytoskeletal elements determines its unique mechanical and dynamic properties.

**Figure 1:**
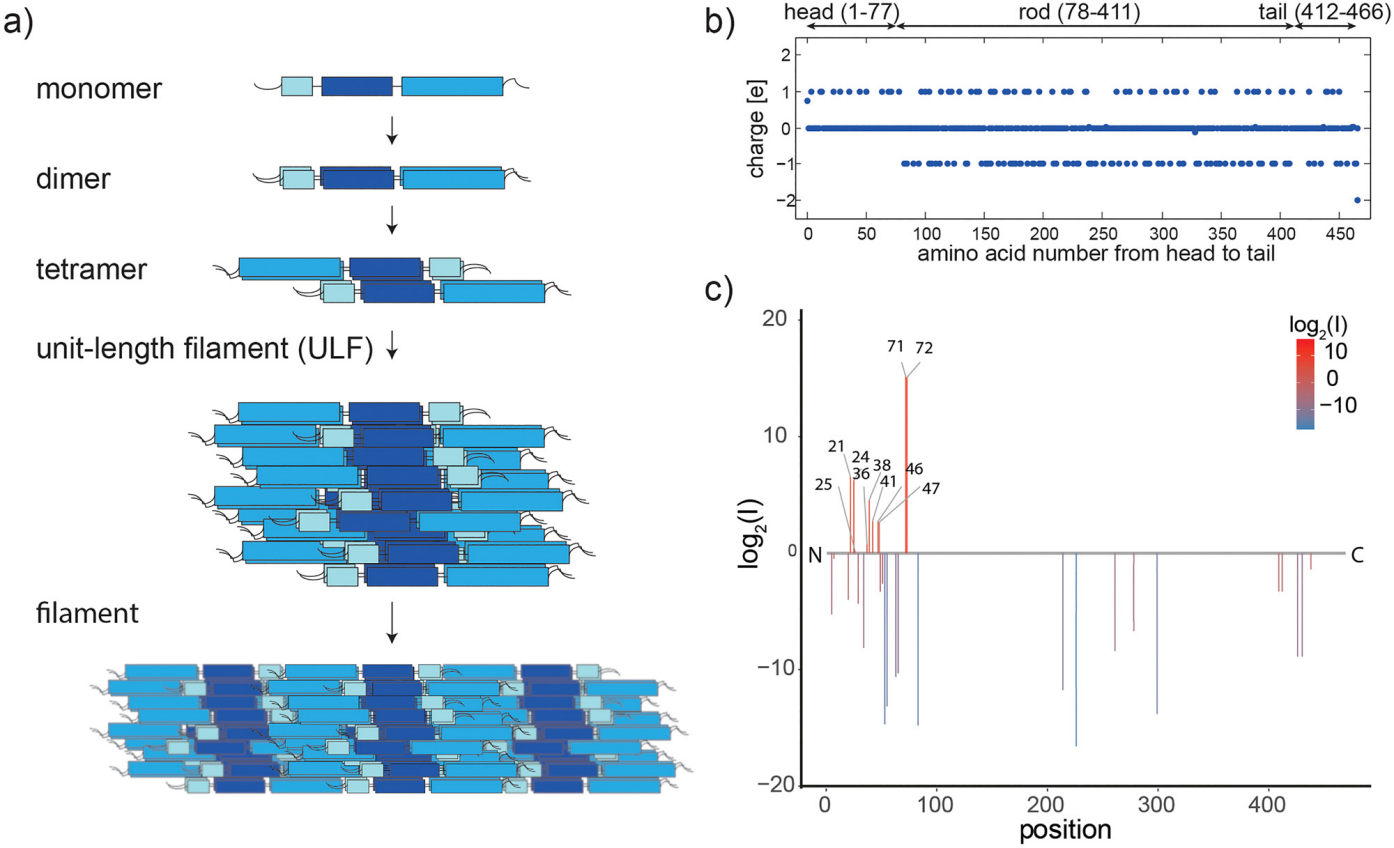
Structural details of intermediate filaments. (a) Assembly pathway of intermediate filaments, shown schematically on the example of vimentin. The monomer consisting of an α-helical rod (colored boxes) and intrinsically disordered head and tail-regions, forms coiled-coil dimers and antiparallel tetramers. Further lateral assembly leads to unit-length filaments and eventually to µm-long filaments (not to scale). (b) The specific amino acids of vimentin determine to a characteristic charge pattern and an overall charge of 19 e^−^ per monomer. (c) Phosphorylated peptides are determined by liquid chromatography mass spectrometry (LC-MS) and the degree of phosphorylation of all identified sites is plotted using the log2 ratios of the LC-MS intensities of phosphorylated over unphosphorylated peptides (intensities *I*). Identified phosphorylation sites and the numbers of amino acids are listed. The color code represents the degree of phosphorylation of all identified phosphorylation sites. Reproduced from Kraxner et al. (2021) with permission from the Royal Society of Chemistry.

Whereas actin and tubulin are conserved across cell types and organisms, IF proteins are expressed in a cell-type specific manner. In humans, more than 70 genes encode for different IF proteins (Szeverenyi et al. 2008), e.g., desmin is found in muscle cells, neurofilaments in neurons and vimentin in cells of mesenchymal origin. IF proteins are grouped into five types according to sequence similarity, and they all share a distinct secondary structure with an α-helical rod domain, flanked by intrinsically disordered head and tail domains (see Figure 1a, top). However, they differ in their amino acid sequence and therefore charge and hydrophobicity patterns along the monomer and consequently along the fully assembled filament. In addition to the cytoplasmic IFs, which are part of the cytoskeleton, lamins (type V IF proteins), form the nuclear lamina at the inside of the nuclear envelope.

Apart from their great variability, another striking characteristic of IFs compared to actin filaments and microtubules is their pronounced stability: whereas actin filaments and microtubules polymerize and depolymerize very dynamically on time scales of seconds to minutes and in a nucleotide-dependent manner, IFs self-assemble in a hierarchical manner and form very stable structures. During mitosis, the cytoplasmic IFs, especially the pronounced, cage-like structure around the nucleus, are disassembled, mediated by posttranslational phosphorylation.

Here, we report on the influence of phosphorylation on IFs and its functions in the cell. We mainly focus on vimentin, which is the most abundant IF in humans (Herrmann et al. 1996; Traub and Vorgias 1983). Moreover, it is best characterized both through experiments on reconstituted systems and in cells. Indeed, vimentin may be regarded as a model for other IFs. The mini-review is structured as follows: first, we summarize the distinct assembly and architecture of IFs. Next, we focus on the charge properties of the filaments and the consequences on intra-filament electrostatic interactions. These considerations then lead us to studies of phosphorylated IFs both in reconstituted systems and in cells, and finally to their influence on the mechanics of IFs.

## 2 Assembly, architecture and mechanics of intermediate filaments

The architecture of IFs is highly hierarchical: the rod-shaped monomers first assemble laterally to coiled-coil dimers, antiparallel tetramers and further to so-called unit-length filaments (ULFs), and then anneal longitudinally into micrometer-long filaments with a diameter of order 10 nm (Herrmann and Aebi 2016), as shown in Figure 1a. The special, open architecture resulting from this assembly pathway renders IFs highly flexible with a persistence length on the order of 1 µm, and extremely extensible at least up to strains of 3.5 (Block et al. 2018, 2017; Forsting et al. 2019; Lorenz et al. 2019). For comparison, actin filaments, which have a similar diameter, but a distinctly different architecture, are about 10 times stiffer and virtually inextensible.

## 3 Electrostatic interactions within intermediate filaments

The sequence of amino acids in the monomer, which is specific to each IF protein, leads to a typical pattern of positive and negative charges (Herrmann and Aebi 2004; Parry and Steinert 1992), which, together with the ionic environment, leads to intra- and interfilament interactions. As an example, the charge pattern is shown for vimentin in Figure 1b. These interactions determine the mechanical properties of the IFs (Schepers et al. 2020) and the interactions between the filaments (Schepers et al. 2021), and can be tuned by changes in the buffer conditions, i.e., ion concentration and pH (Schepers et al. 2020). A further possibility for a cellular system to add charges to a protein is phosphorylation of certain amino acids, notably serine the threonine, and indeed, vimentin possesses a high number of phosphorylation sites (Hyder et al. 2008; Snider and Omary 2014) as shown in Figure 1c (Kraxner et al. 2021)**.** These phosphorlyations are mediated by a variety of kinases like protein kinase A and C (PKA, PKC) and most phosphorylation sites are found in the head region of vimentin (Eriksson et al. 2004). Phosphorylation at specific sites of vimentin leads to disassembly, which is a necessary prerequisite for cell division during mitosis (Eriksson et al. 1992; Inagaki et al. 1996).

## 4 Phosphorylation of intermediate filaments in reconstituted systems

As mentioned above, vimentin is likely the best-characterized IF protein in reconstituted systems. Consequently, an assay to study the influence of phosphorylations on the assembly and disassembly dynamics of vimentin was established early on (Inagaki et al. 1987), employing purified vimentin from cells and several different purified protein kinases. The authors found that phosphorylation with either PKC or PKA interferes strongly with the assembly into normal filaments. Note that in earlier literature the name “catalytic subunit of cAMP-dependent protein kinase” was used as a synonym for PKA. Interestingly, PKA incorporates more phosphate into assembled filaments (0.8 mol per mole of vimentin compared to 0.5 mol for PKC), but less into monomeric vimentin (1.3 mol per mol of vimentin compared to 2.5 mol for PKC). As the phosphorylation sites differ for these two kinases, this indicates that PKC is in general more effective in phosphorylating vimentin, but in the fully assembled vimentin IF, the specific sites, which PKA acts on, are more accessible. PKA acts in a more pronounced manner on the sites that lead to more dramatic disassembly. In general, phosphorylation by these different kinases is site-specific, i.e., the specific amino acids which are phosphorylated differ according to the respective kinase.

**Figure 2:**
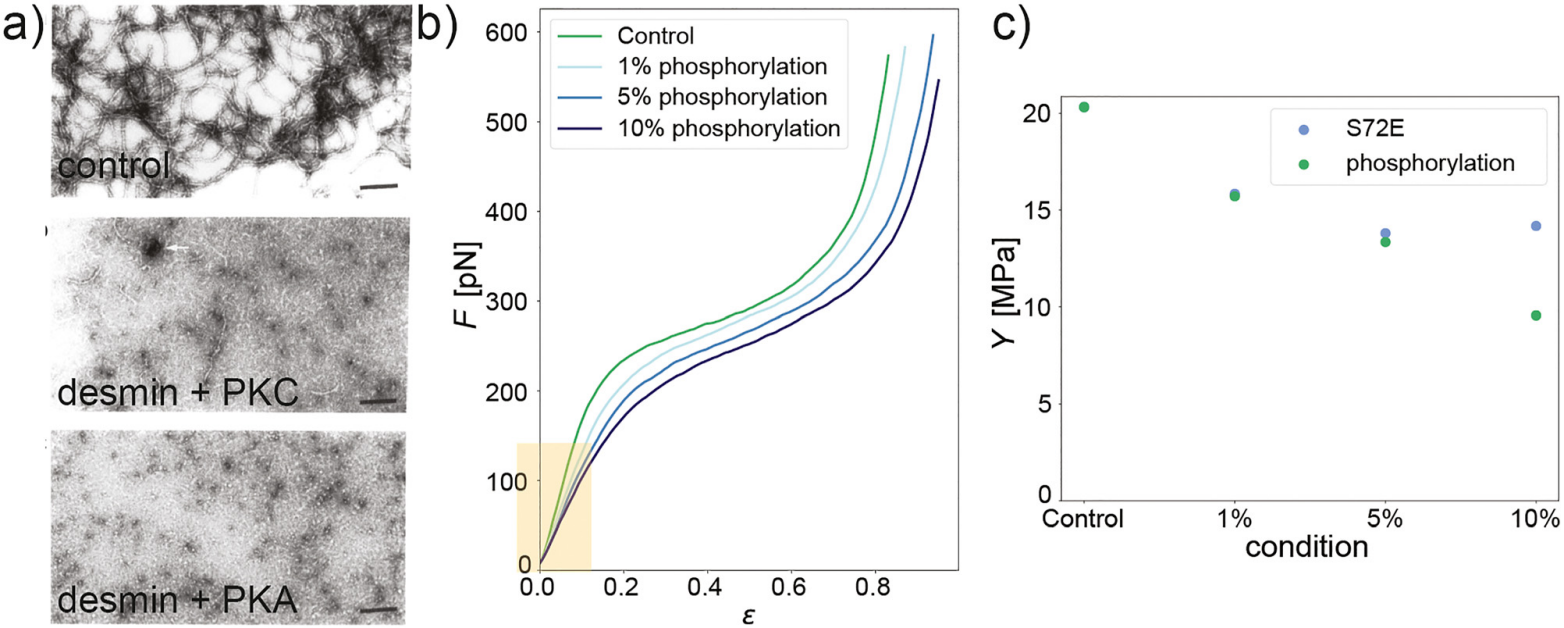
Phosphorylation of intermediate filaments in reconstituted systems. (a) Electron micrographs (negative staining) of normally assembled desmin filaments (top) in comparison to filaments phosphorylated by PKC (center) and PKA (bottom). Adapted from Inagaki et al. (1988). (b) Force strain data from optical tweezer experiments, showing that the stiffness decreases upon incorporation of phosphorylated vimentin (see slope of the curves for small strains, yellow box). (c) Young’s modulus *Y* as derived from the slope at small strains (see panel b); in comparison for vimentin phosphorylated by PKA and the phosphomimetic vimentin mutant S72E. Panels (b) and (c) adapted from Kraxner et al. (2021) with permission from the Royal Society of Chemistry.

The same assay was then also applied to desmin, the IF protein found in muscle cells. Figure 2a shows electron micrographs of desmin assembled in the absence of kinases (top), with PKC (center) and with PKA (bottom) (Inagaki et al. 1988). Compared to the control without kinase, phosphorylation leads to the formation of aggregates (Geisler and Weber 1988). Like vimentin, desmin belongs to the type III IF proteins and it is thus not surprising that the two proteins show similarities concerning their assembly/disassembly pathways. Site specific kinases leading to the disassembly of IFs were identified for a large number of IF proteins (see (Inagaki et al. 1996) and references therein) and interestingly, despite the large similarities between the different IF proteins, each of them is associated with a subgroup of kinases, which mediates disassembly. From these early studies it was concluded that the phosphorylation of IF proteins is used by the cell to regulate the reversible assembly of cytoskeletal filaments, which are, in turn, important in various cell functions.

The most prominent of such functions is mitosis: the stable IF network, which among other structures forms a “cage” around the nucleus, needs to be disassembled for the cell to be able to divide. In a combined study on cells and reconstituted filaments the authors could show, in a time-resolved manner, how filaments disassemble during the time-course of 40 min (Chou et al. 1989). Notably, this process is only observed, when protein kinase and ATP are present, thus underlining the necessity of the presence of an active kinase. Interestingly, in both vimentin and desmin the preferred phosphorylation sites lie in the head domain (Geisler et al. 1989; Geisler and Weber 1988) that is also crucial for filament assembly as shown in experiments with headless vimentin, which is unable to assemble (Rogers et al. 1995).

## 5 Phosphorylation of intermediate filaments in cells

**Figure 3:**
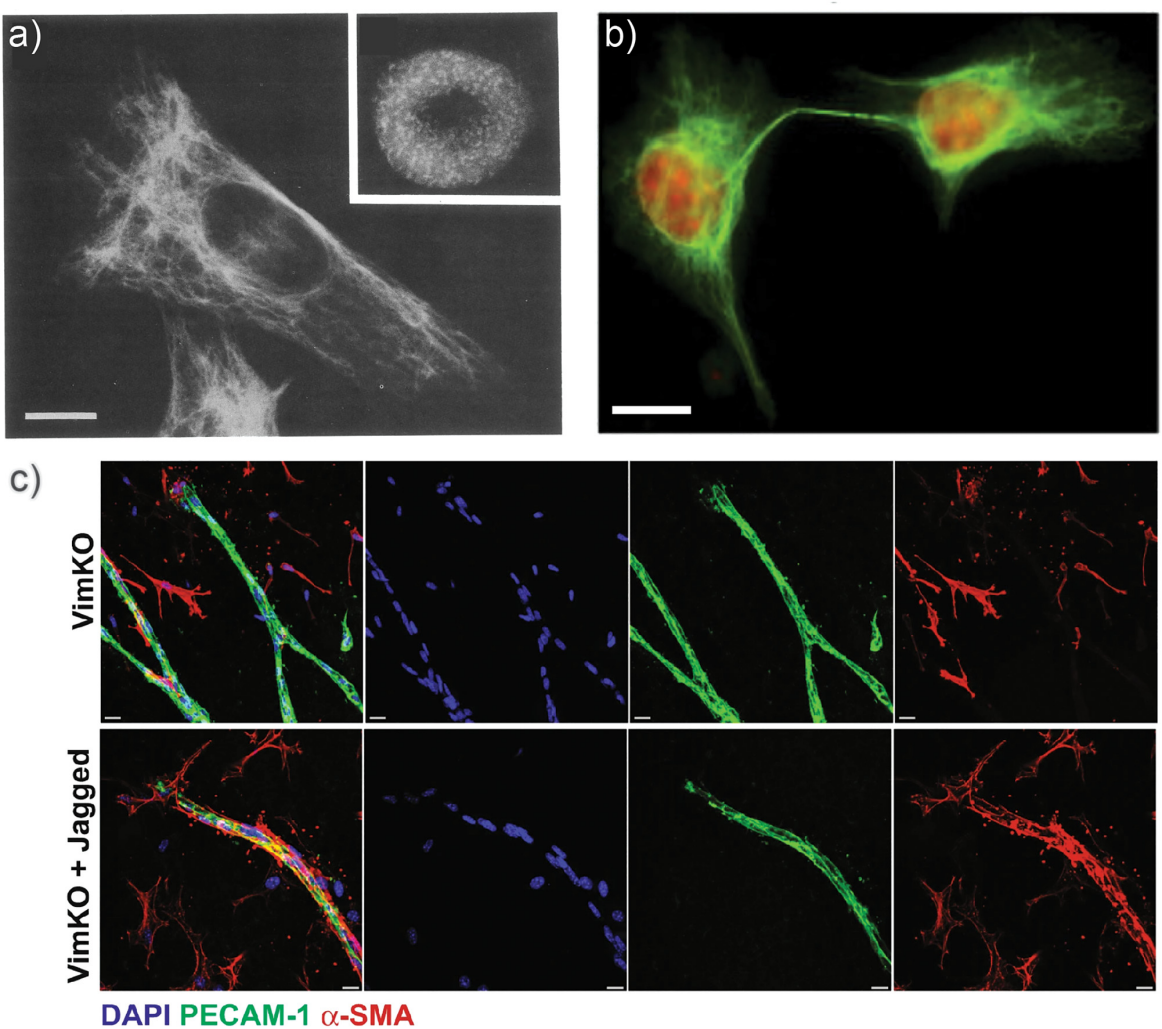
Phosphorylation of intermediate filaments in cells. (a) Fluorescence images of immunostained (desmin/vimentin) BHK-21 cells. The main image shows the intact IF network in cells during interphase and the inset (top right) show the disassembled IF network during metaphase. Adapted from Chou et al. (1989). The scale bar is 10 µm. (b) Immunostained urinary bladder epithelial cancer cells (vimentin in green; propidium iodide to visualize the nuclei in red): IF bridge between two daughter cells which results from a mutation in Ser72 that inhibits phosphorylation at that position. Adapted from Sihag et al. (2007). The scale bar is 10 µm. (c) Role of vimentin in regulating vascular smooth muscle cell (VSMC) coverage of aortic rings through the Notch ligand Jagged1: Loss of vimentin leads to significantly reduced VSMC coverage (upper panel, red staining) and changes in the mechanical properties of artery. Reactivation of Jagged1 rescues the phenotype of VSMC coverage of the aortic rings (lower panel, red staining). Jagged1 interacts with vimentin in its phosphorylated state (Ser38) and phosphorylation is increased by shear stress. Red: α-smooth muscle actin (α-SMA), green: PECAM-1, a protein found in intracellular junctions and a marker for endothelial cells; blue: DNA. Adapted from van Engeland et al. (2019). The scale bars represent 10 µm.

Many, although not all (Inagaki et al. 1996), kinases that phosphorylate IFs in reconstituted systems are also active in the cell. Indeed, in living cells, phosphorylation of IFs is a crucial prerequisite for a number of scenarios. A very prominent example is the rapid disassembly of the IF network during mitosis (Chou et al. 1989): The tight cage of cytoplasmic IFs located around the nucleus, as shown in Figure 3a, needs to be resolved before cell division and reconstituted thereafter. Early studies revealed that phosphorylation of vimentin at Ser55 by p34^cdc2^ kinase is responsible for the disassembly during mitosis (Chou et al. 1990), and later studies identified additional interphase-specific phosphorylation sites (Sihag et al. 2007). As another requirement for successful cell division and simultaneously with the disassembly of the vimentin network, nuclear lamins become hyperphosphorylated leading to a breakdown of the nuclear envelope (Chou et al. 1990). Interestingly, during mitosis vimentin is mainly phosphorylated in the head domain (Chou et al. 1989), in line with the importance of this domain for filament assembly (Rogers et al. 1995). These phosphorylation events result in the release of tetrameric vimentin subunits to the cytoplasm (Eriksson et al. 2004). More recent studies revealed a sophisticated spatio-temporal regulation of the phosphorylation process during mitosis: first, Cdk1 phosphorylates vimentin from prometaphase to metaphase, and subsequently, Aurora-B and Rho-kinase phosphorylate vimentin from anaphase to the end of mitosis (Sihag et al. 2007). Consequently, mutations at phosphorylation sites or abnormal regulation of mitotic kinases lead to various problems. For example, a mutation at Ser72 in vimentin leads to IF bridges between two daughter cells as shown in Figure 3b. In general, errors in kinase regulation result in the generation of tetraploid cells with two nuclei, which leads to cell death under physiological conditions (Sihag et al. 2007). A deeper understanding of these abnormal kinase regulations could provide insights in aneuploid cells in cancer development.

Beyond regulation of the solubility of IFs, as in the case of, e.g., vimentin during cell division, phosphorylation serves additional functions in living cells. Neurofilaments are found in neurons and consist of three proteins that differ concerning the length of their tail domain (Herrmann and Aebi 2004). Like vimentin, all three neurofilament proteins are hyperphosphorylated after their assembly into filaments, which are transported into the axon in a phosphorylation dependent manner (Sihag et al. 2007) but unlike for vimentin, this does not lead to disassembly. By contrast, phosphorylated neurofilaments are found in postmitotic neurons, which do not divide, i.e., the filaments do not undergo depolymerization anymore. Interestingly, in neurofilament proteins, the tail domains are highly phosphorylated. After assembly, they decorate the surface of the filament. In this context, a correlation between neurofilament phosphorylation and axonal transport rates was found (Sihag et al. 2007), and it was concluded that the phosphorylation affects binding affinity to other proteins, such as microtubules. Furthermore, a relation between vimentin phosphorylation and Notch signaling, which in response to shear stress by the blood flow regulates arterial remodeling via the signaling between endothelial cells (ECs) and vascular smooth muscle cells (VSMCs), was found (see Figure 3c) (van Engeland et al. 2019).

## 6 Influence of phosphorylation on the mechanics of intermediate filaments

As discussed above, when fully, or at least to a large extent, phosphorylated, IFs disassemble and phosphorylated tetramers do not reassemble. In the intermediate regime, i.e., when phosphorylated and non-phosphorylated protein is mixed within one filament, the protein structures soften. We have systematically investigated this phenomenon by directly measuring the stiffness of reconstituted filaments using an optical tweezer pulling assay (Kraxner et al. 2021). In force-strain curves, as shown in Figure 2b, the slope for small strains, i.e., in the linear regime (see yellow shaded box), corresponds to the stiffness of the filaments and is used to derive the Young’s modulus (Figure 2c). PKA-treated protein, as well as the phosphomimetic mutant S72E show a more pronounced softening the more of the phosphorylated or mutant protein is incorporated in the filaments.

Within living cells, the consequence of such softening may be the ability to locally and temporarily tune the mechanical properties of the IF cytoskeleton in a very precise way. Such adaptability via phosphorylation likely plays a role in situations where the cell migrates and moves through small constrictions, such as in immune response, cancer metastasis or wound healing and indeed, phosphorylation of vimentin enhances migration in cells (Chung et al. 2013; Snider and Omary 2014). A prominent example is the migration of leucocytes to inflammation sites, which depends on vimentin phosphorylation. Vimentin is a downstream effector of the phosphoinositide 3-kinase γ (PI3Kγ) signaling pathway in macrophages and it was shown that PI3Kγ-dependent signaling is essential for the remodeling of the cytoskeleton which in turn is necessary for macrophages to migrate (Barberis et al. 2009). The knock-out of PI3Kγ results in a reduced phosphorylation of vimentin and in an impaired migration of macrophages. However, transfection with a vimentin mutant that mimics phosphorylation is able to rescue the trans-endothelial migration defect induced by the knock-out. Thus, this experiment directly links the necessity of vimentin phosphorylation with cellular migration.

Vimentin was found to be a target of the PI3K/AKT signaling pathway which plays important roles in a variety of cancer types (Zhu et al. 2011). The binding of AKT to vimentin leads to phosphorylation of Ser39 which results in an increase in mobility and invasion, again relating vimentin phosphorylation to an increased migration ability. An alanine mutation at the phosphorylation site Ser39, such that this site cannot be phosphorylated anymore, results in a disturbed ability of vimentin to induce migration and invasion, reinforcing the importance of this specific phosphorylation site (Zhu et al. 2011).

## 7 Conclusions

Taken together, phosphorylation of IF proteins does not solely provide a mechanism for reversible assembly of filaments but rather offers a huge variety of regulation mechanisms ranging from remodeling of IF networks (Chou et al. 1989; Eriksson et al. 2004), cell migration (Chung et al. 2013), changes in mechanical properties (Kraxner et al. 2021), interactions with other protein structures (Sihag et al. 2007) or roles in signaling pathways (van Engeland et al. 2019). Nevertheless, a significant part of the function of specific phosphorylation sites in IFs is yet unknown and needs further investigation. Future experiments could investigate which sites of vimentin are phosphorylated under different scenarios or stress situations and try to map the function of each of these sites.

## References

[j_hsz-2023-0140_ref_001] Barberis L., Pasquali C., Bertschy-Meier D., Cuccurullo A., Costa C., Ambrogio C., Vilbois F., Chiarle R., Wymann M., Altruda F. (2009). Leukocyte transmigration is modulated by chemokine-mediated PI3Kγ-dependent phosphorylation of vimentin. Eur. J. Immunol..

[j_hsz-2023-0140_ref_002] Block J., Witt H., Candelli A., Danes J.C., Peterman E.J.G., Wuite G.J.L., Janshoff A., Köster S. (2018). Viscoelastic properties of vimentin originate from nonequilibrium conformational changes. Sci. Adv..

[j_hsz-2023-0140_ref_003] Block J., Witt H., Candelli A., Peterman E.J.G., Wuite G.J.L., Janshoff A., Köster S. (2017). Nonlinear loading-rate-dependent force response of individual vimentin intermediate filaments to applied strain. Phys. Rev. Lett..

[j_hsz-2023-0140_ref_004] Chou R.G.R., Stromer M.H., Robson R.M., Huiatt T.W. (1990). Determination of the critical concentration required for desmin assembly. Biochem. J..

[j_hsz-2023-0140_ref_005] Chou Y.H., Rosevear E., Goldman R.D. (1989). Phosphorylation and disassembly of intermediate filaments in mitotic cells. Proc. Natl. Acad. Sci. U. S. A..

[j_hsz-2023-0140_ref_006] Chung B.-M., Rotty J.D., Coulombe P.A. (2013). Networking galore: intermediate filaments and cell migration. Curr. Opin. Cell Biol..

[j_hsz-2023-0140_ref_007] Eriksson J.E., Brautigan D.L., Vallee R., Olmsted J., Fujiki H., Goldman R.D. (1992). Cytoskeletal integrity in interphase cells requires protein phosphatase activity. Proc. Natl. Acad. Sci. U. S. A..

[j_hsz-2023-0140_ref_008] Eriksson J.E., He T., Trejo-Skalli A.V., Härmälä-Braskén A.-S., Hellman J., Chou Y.-H., Goldman R.D. (2004). Specific in vivo phosphorylation sites determine the assembly dynamics of vimentin intermediate filaments. J. Cell Sci..

[j_hsz-2023-0140_ref_009] Forsting J., Kraxner J., Witt H., Janshoff A., Köster S. (2019). Vimentin intermediate filaments undergo irreversible conformational changes during cyclic loading. Nano Lett..

[j_hsz-2023-0140_ref_010] Geisler N., Hatzfeld M., Weber K. (1989). Phosphorylation in vitro of vimentin by protein kinases A and C is restricted to the head domain. Identification of the phosphoserine sites and their influence on filament formation. Eur. J. Biochem..

[j_hsz-2023-0140_ref_011] Geisler N., Weber K. (1988). Phosphorylation of desmin *in vitro* inhibits formation of intermediate filaments; identification of three kinase A sites in the aminoterminal head domain. EMBO J..

[j_hsz-2023-0140_ref_012] Herrmann H., Aebi U. (2016). Intermediate filaments: structure and assembly. Cold Spring Harb. Perspect. Biol..

[j_hsz-2023-0140_ref_013] Herrmann H., Aebi U. (2004). Intermediate filaments: molecular structure, assembly mechanism, and integration into functionally distinct intracellular scaffolds. Annu. Rev. Biochem..

[j_hsz-2023-0140_ref_014] Herrmann H., Häner M., Brettel M., Müller S.A., Goldie K.N., Fedtke B., Lustig A., Franke W.W., Aebi U. (1996). Structure and assembly properties of the intermediate filament protein vimentin: the role of its head, rod and tail domains. J. Mol. Biol..

[j_hsz-2023-0140_ref_015] Huber F., Boire A., López M.P., Koenderink G.H. (2015). Cytoskeletal crosstalk: when three different personalities team up. Curr. Opin. Cell Biol..

[j_hsz-2023-0140_ref_016] Hyder C.L., Pallari H.-M., Kochin V., Eriksson J.E. (2008). Providing cellular signposts – post-translational modifications of intermediate filaments. FEBS Lett..

[j_hsz-2023-0140_ref_017] Inagaki M., Gonda Y., Matsuyama M., Nishizawa K., Nishi Y., Sato C. (1988). Intermediate filament reconstitution *in vitro*. The role of phosphorylation on the assembly-disassembly of desmin. J. Biol. Chem..

[j_hsz-2023-0140_ref_018] Inagaki M., Matsuoka Y., Tsujimura K., Ando S., Tokui T., Takahashi T., Inagaki N. (1996). Dynamic property of intermediate filaments: regulation by phosphorylation. BioEssays.

[j_hsz-2023-0140_ref_019] Inagaki M., Nishi Y., Nishizawa K., Matsuyama M., Sato C. (1987). Site-specific phosphorylation induces disassembly of vimentin filaments *in vitro*. Nature.

[j_hsz-2023-0140_ref_020] Kraxner J., Lorenz C., Menzel J., Parfentev I., Silbern I., Denz M., Urlaub H., Schwappach B., Köster S. (2021). Post-translational modifications soften vimentin intermediate filaments. Nanoscale.

[j_hsz-2023-0140_ref_021] Lorenz C., Forsting J., Schepers A.V., Kraxner J., Bauch S., Witt H., Klumpp S., Köster S. (2019). Lateral subunit coupling determines intermediate filament mechanics. Phys. Rev. Lett..

[j_hsz-2023-0140_ref_022] Parry D.A.D., Steinert P.M. (1992). Intermediate filament structure. Curr. Opin. Cell Biol..

[j_hsz-2023-0140_ref_023] Rogers K.R., Eckelt A., Nimmrich V., Janssen K.P., Schliwa M., Herrmann H., Franke W.W. (1995). Truncation mutagenesis of the non-α-helical carboxyterminal tail domain of vimentin reveals contributions to cellular localization but not to filament assembly. Eur. J. Cell Biol..

[j_hsz-2023-0140_ref_024] Schepers A.V., Lorenz C., Köster S. (2020). Tuning intermediate filament mechanics by variation of ph and ion charges. Nanoscale.

[j_hsz-2023-0140_ref_025] Schepers A.V., Lorenz C., Nietmann P., Janshoff A., Klumpp S., Köster S. (2021). Multiscale mechanics and temporal evolution of vimentin intermediate filament networks. Proc. Natl. Acad. Sci. U. S. A..

[j_hsz-2023-0140_ref_026] Sihag R.K., Inagaki M., Yamaguchi T., Shea T.B., Pant H.C. (2007). Role of phosphorylation on the structural dynamics and function of types III and IV intermediate filaments. Exp. Cell Res..

[j_hsz-2023-0140_ref_027] Snider N.T., Omary M.B. (2014). Post-translational modifications of intermediate filament proteins: mechanisms and functions. Nat. Rev. Mol. Cell Biol..

[j_hsz-2023-0140_ref_028] Szeverenyi I., Cassidy A.J., Chung C.W., Lee B.T.K., Common J.E.A., Ogg S.C., Chen H., Sim S.Y., Goh W.L.P., Ng K.W. (2008). The Human Intermediate Filament Database: comprehensive information on a gene family involved in many human diseases. Hum. Mutat..

[j_hsz-2023-0140_ref_029] Traub P., Vorgias C.E. (1983). Involvement of the N-terminal polypeptide of vimentin in the formation of intermediate filaments. J. Cell Sci..

[j_hsz-2023-0140_ref_030] van Engeland N.C.A., Suarez Rodriguez F., Rivero-Müller A., Ristori T., Duran C.L., Stassen O.M.J.A., Antfolk D., Driessen R.C.H., Ruohonen S., Ruohonen S.T. (2019). Vimentin regulates Notch signaling strength and arterial remodeling in response to hemodynamic stress. Sci. Rep..

[j_hsz-2023-0140_ref_031] Zhu Q.-S., Rosenblatt K., Huang K.-L., Lahat G., Brobey R., Bolshakov S., Nguyen T., Ding Z., Belousov R., Bill K. (2011). Vimentin is a novel AKT1 target mediating motility and invasion. Oncogene.

